# The Temporal Dynamics of Voluntary Emotion Regulation

**DOI:** 10.1371/journal.pone.0006726

**Published:** 2009-08-25

**Authors:** Henrik Walter, Alexander von Kalckreuth, Dina Schardt, Achim Stephan, Thomas Goschke, Susanne Erk

**Affiliations:** 1 Division of Medical Psychology, University of Bonn, Bonn, Germany; 2 Department of Psychiatry and Psychotherapy, University of Bonn, Bonn, Germany; 3 Department of Psychiatry III, University of Ulm, Ulm, Germany; 4 Department of Neurology, Technische Universitaet Muenchen, Munich, Germany; 5 Institute of Neuroradiology, Hannover Medical School, Hannover, Germany; 6 Institute of Cognitive Science, University of Osnabrueck, Osnabrueck, Germany; 7 Department of Psychology, Technical University Dresden, Dresden, Germany; University of Groningen, Netherlands

## Abstract

**Background:**

Neuroimaging has demonstrated that voluntary emotion regulation is effective in reducing amygdala activation to aversive stimuli *during* regulation. However, to date little is known about the sustainability of these neural effects once active emotion regulation has been terminated.

**Methodology/Principal Findings:**

We addressed this issue by means of functional magnetic resonance imaging (fMRI) in healthy female subjects. We performed an active emotion regulation task using aversive visual scenes (task 1) and a subsequent passive viewing task using the same stimuli (task 2). Here we demonstrate not only a significantly reduced amygdala activation during active regulation but also a sustained regulation effect on the amygdala in the subsequent passive viewing task. This effect was related to an immediate increase of amygdala signal in task 1 once active emotion regulation has been terminated: The larger this peak postregulation signal in the amygdala in task 1, the smaller the sustained regulation effect in task 2.

**Conclusions/Significance:**

In summary, we found clear evidence that effects of voluntary emotion regulation extend beyond the period of active regulation. These findings are of importance for the understanding of emotion regulation in general, for disorders of emotion regulation and for psychotherapeutic interventions.

## Introduction

In the last decade cognitive neuroscience has adopted emotions as a subject of research resulting in the development of the field of affective neuroscience. One important part of emotion research is emotion regulation. Actually, one of the pioneers of emotion psychology has argued that emotion regulation is part and parcel of emotion itself [1]. Moreover, dysfunction of emotion regulation is central to psychiatric conditions, in particular affective and anxiety disorders [2]. A recent review has highlighted the difference between automatic and voluntary emotion regulation [3]. Automatic emotion regulation refers to all forms of change in emotion processing which happen implicitly and without conscious intention. In contrast, voluntary emotion regulation refers to effortful and controlled processes based on consciously intended strategies, and is subject of the present report.

Following early works on anxiety regulation in psychoanalysis [4] as well as early work of Lazarus on coping [5], a number of contemporary behavioral studies has demonstrated that cognitive emotion regulation can reduce negative feelings by re-appraising an unpleasant situation in less emotional terms indexed by reduced autonomic and startle responses (e.g. [6], [7]).

In the last several years, cognitive neuroscience has shown that voluntary emotion regulation is effective in reducing negative feelings and corresponding physiological responses in the amygdala [3], [8], [9]. Neuroimaging studies have investigated various strategies such as labelling [10], distraction [11], [12], detachment [13]–[15] or reappraising a negative event in unemotional terms [16]–[18]. The brain network active during regulation of negative affect comprises medial and lateral prefrontal areas as well as the parietal cortex [3], [10], [13]–[21]. It has been proposed that prefrontal regions exert a top-down inhibitory effect on the amygdala, shown in a negative correlation between the ventrolateral [17] or the ventromedial prefrontal cortex [22] and the amygdala. Connectivity analyses have shown reappraisal-dependent coupling between the amygdala and specific regions in the prefrontal cortex such as dorsolateral and -medial PFC, orbitofrontal cortex as well as the subgenual anterior cingulate cortex [23]. Voluntary emotion regulation has also been investigated in patient populations such as those with depression [24], [25] or social anxiety disorder [26], showing a dysfunction of the cortico-limbic circuit.

Although these studies show that emotion regulation is effective on the behavioural and neural level during active regulation, we still do not know whether these effects extend beyond the regulation period itself. For example, what happens to brain activation in the amygdala, the key structure in processing negative emotions, after successful regulation of a negative emotion? None of the above mentioned studies has investigated this explicitly. Emotion regulation may in fact have sustained downregulatory effects on the amygdala. However, emotion regulation might also result in a paradoxical increase of amygdala activation after termination of voluntary emotion regulation. Rebound of emotions has been a central topic in the early days of psychology as ‘the return of the repressed’ [27]. In empirical psychology behavioral emotional rebound effects have been described in the context of thought suppression [28]–[31].

In contrast to studies on thought suppression we focused on a cognitive strategy of emotion regulation known as detachment, which has been shown to be effective in momentary emotion reduction and amygdala downregulation [14], [19], [21]. Specifically, we were interested in amygdala activation as a neural signature of emotion processing *after termination* of the intentional effort to regulate. We explored whether there would be sustained downregulation or a postregulational, paradoxical increase of amygdala activation by studying the temporal dynamics of amygdala activation in two sequenced tasks. In the first task we investigated amygdala downregulation during active emotion regulation, as well as the signal time course within the amygdala immediately after termination of voluntary emotion regulation. In the second task, completed by the same subjects approximately ten minutes after the first task, we studied amygdala activation during a passive viewing paradigm with the same stimuli. Thus, we were able to test for sustained or paradoxical effects of emotion regulation on two different time scales.

## Methods

### Ethics statement

The protocol was approved by the ethics committee of the University of Ulm. All subjects gave written informed consent.

### Subjects and Task

20 healthy right-handed female volunteers (24±3 years) without any history of neurological or psychiatric illness participated in the imaging study. Participants were instructed carefully and trained with some examples before the scanning session with the opportunity to ask if they would have difficulties in understanding the instructions. However, all subjects understood and could follow the instructions without difficulty as seen in the debriefing. Subjects completed versions of the Beck Depression Inventory (BDI), the State Trait Anxiety Inventory (STAI) and the Toronto Alexithymia Scale (TAS-20) in order to rule out effects of altered mood states and deficits in emotion perception. Two subjects reached BDI scores above the cut-off score of 9 and thus were discarded from further analyses. Subjects also completed the Emotion Regulation Questionnaire [32] in order to test for the influence of habitual emotion regulation strategies on potential aftereffects. Finally subjects completed the White Bear Suppression Inventory (WBSI), a measure of habitual thought control, which has been shown to correlate with a postsuppressional rebound [33]. Both scores, the ERQ and the WBSI, were obtained in order to test whether they modulated potential aftereffects of emotion regulation.

The study was composed of two tasks. During task 1 (“active regulation”), subjects saw 60 pictures of negative or neutral content taken from the International Affective Picture System and matched for complexity and content of faces, scenery, food and nature (mean valence (V) and arousal (A) values: negative no regulation: V = 2.7, A = 5.4, negative regulation: V = 2.8, A = 5.4, neutral no regulation: V = 5.7, A = 3.4, neutral regulation: V = 5.7, A = 3.2). Subjects were instructed to either simply watch the pictures and permit all upcoming emotions or to intentionally regulate their emotions by taking the position of a neutral observer. More specifically they were instructed to: “Look at the following picture directly but try to take the position of a noninvolved observer, thinking about the present picture in a neutral way” for the regulation condition or “Look at the following picture directly and permit feeling your emotions” for the no-regulation condition. The instruction during scanning was given by presenting a cue word for 2 s stating either “permit” or “regulate”. After picture presentation for 8 s, subjects were instructed to not regulate any more and relax. Duration of this relaxation period was 20 s. Trials were presented in randomized order. Task 1 was performed in two consecutive sessions of 15 minutes each.

Approximately ten minutes after end of task 1, we tested for a delayed aftereffect in task 2 (“passive viewing”). Subjects were instructed to just look at the 60 pictures from the IAPS again which were presented for only 1 s each in a newly randomized order. Thus, we minimized intentional emotion regulation efforts. Intertrial interval was 3 s with a variable jitter within±1 TR.

SInce it has been shown that even the linguistic evaluation of emotional stimuli can significantly reduce amygdala activation [10], [34], we intentionally refrained from a trial-by-trial rating of emotional intensity and evaluated regulation success post-hoc, thereby avoiding influencing amygdala activation after emotion regulation termination. However, we additionally performed a control experiment in an independent sample (n = 10) using a trial-by-trial rating in order to confirm the success of emotion regulation of our procedure.

### Functional Imaging

Imaging was performed on a 3 Tesla Siemens Allegra scanner equipped with a head coil. T2* weighted functional MR images were obtained using event-related echoplanar imaging in a tangential-axial orientation (using an imaginary line from the orbitofrontal cortex to the cerebellum) in order to minimize susceptibility artifacts. Image size was 64×64 pixels, with a field of view of 192 mm, flip angle was 90°. In task 1, one volume covering the whole brain consisted of 31 slices. Slice thickness was 3 mm with 25% gap resulting in a voxel size of 3×3×3.75 mm. Volumes were obtained every 2000 ms (TE 35 ms). In task 2 volumes were obtained every 1500 ms (TE 35 ms). One volume consists of 23 slices with a slice thickness of 3 mm with 25% gap, covering the brain from the temporal poles to the superior parietal cortex and thus in each case included the amygdala, the prefrontal and the parietal cortex. All other acquisition parameters were similar to task 1. Stimuli were presented with LCD video goggles (Resonance Technologies, Northridge, CA). For each subject, three-dimensional T1 weighted anatomical volumes were acquired.

### Data Analysis

Data preprocessing and statistical analyses were carried out with SPM 2 (http://www.fil.ion.ucl.ac.uk/spm/software/spm2/) and Matlab 6.5.1 (MathWorks, Natick, MA) and were similar for both tasks. Preprocessing included realignment, spatial normaliziation to the EPI-template (2×2×2 mm) and spatial smoothing (8 mm). For each trial the variance of each voxel was estimated according to the General Linear Model. Intrinsic autocorrelations were accounted for by an autoregressive model of 1^st^ order and low frequency drifts were removed via high pass filter.

The regression model consisted of a set of 5 regressors (instruction, non-regulated negative, non-regulated neutral, regulated negative, regulated neutral) convolved with the hemodynamic response function and six regressors describing residual motion. In a second level random effects group analysis, individual regionally specific effects of conditions for each subject were compared using a within-subject ANOVA (2×2 design with the factors regulation condition (regulation, no regulation) and valence (negative, neutral) with non-sphericity correction resulting in a *t*-statistic for every voxel. *T*-statistics for each voxel were thresholded at p<0.05 corrected for multiple comparisons across whole brain with a family wise error rate (FWE). For the amygdala, which was our region of interest, we corrected for multiple comparisons within an anatomically defined region of interest [35] at p<0.05, FWE corrected.

In order to test for an immediate aftereffect in task 1 we extracted for each subject the averaged 1^st^ eigenvariate time series centered around the amygdala voxel displaying peak activity in the group analysis for the main effect of emotion (left x = −22, y = −2, z = −24; right x = 18, y = 0, z = −20) using an 8-mm radial sphere. We calculated the mean time course for each subject and each condition and included data in an ANOVA for repeated measures with the factors valence, regulation and period (Statistica 6.0, Tulsa, OK, USA). Data were taken from scan 5 (i.e. 6 s after picture onset) and scan 9 (i.e. 6 sec after picture offset), where the hemodynamic signal related to picture presentation and relax period is proposed to reach its peak. Inspection of the individual time courses confirmed this procedure.

## Results

### Behavioral results

Emotion regulation was successful as rated post-hoc on a 9-point-Likert scale with mean = 3.39 (SD = 1.0) (1 = very, 9 = not successful). This was confirmed in a control experiment in an independent sample (n = 10), using a trial-by-trial rating of subjective negative affect (0 = weakest, 7 = strongest) exactly as in [36]: negative affect was significantly lower for regulated negative trials (3.18 (0.49)) compared to non-regulated negative trials (5.71 (0.49); F(1,9) = 56.3; p = 0.000037).

All 18 subjects included in the analyses had average (non pathological) scores on the BDI, STAI and TAS-20 (BDI (mean: 3; range: 1–9), STAI-S (34.5; 26–49),STAI-T (33; 24–50), TAS-20 (48; 37–65). Mean (range) score for the WBSI was 32.5 (18–67), for the suppression subscale of the ERQ (ERQ-S) 12 (6–26) and the reappraisal subscale (ERQ-R) 30 (18–39).

### fMRI Data

**Figure 1:**
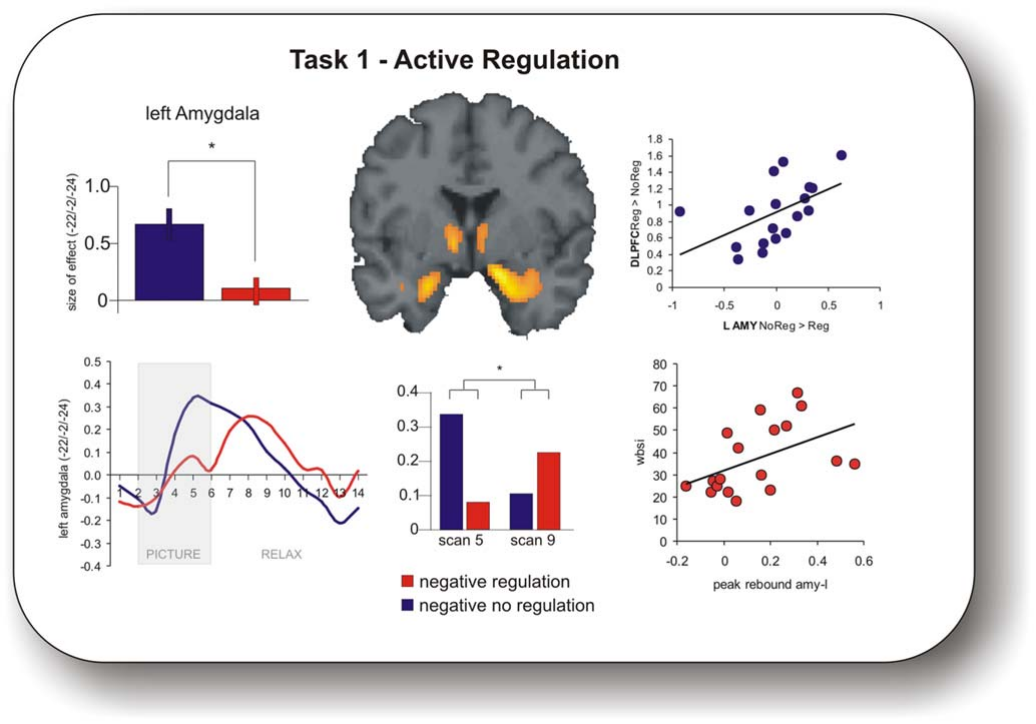
Task 1 (active regulation). Upper row left: Amygdala activation was significantly attenuated during regulation (p<0.05 FWE corrected for ROI). This regulation related decrease of amygdala activation was positively correlated with a regulation related increase in DLPFC activation (Upper row, right). Bottom row, left: Time course of left Amygdala, showing a significant postregulation rebound and a significant interaction of regulation and period (bar plot bottom row, middle). Note: all effects here shown for the left amygdala, are also significant for the right amygdala. Bottom row, right: Positive correlation between peak activation during relax period (rebound) in left amygdala and individual scores in the WBSI.

*Task 1 (active regulation):* For detailed results please see Table 1. During regulation we observed a significant activation of a prefronto-parietal network, comprising dorsolateral prefrontal and inferior parietal cortices. Importantly, the main effect of valence revealed bilateral amygdala activation which was significantly reduced during regulation (Fig. 1, Tab. 1).

**Table 1:** Results of Task 1 (Active Regulation).

Region		BA	Z	x	y	z
*Group activation for negative>neutral*						
dorsolateral prefrontal cortex	R	46	5.42	48	34	10
inferior frontal gyrus	L	44	4.97	−42	4	28
	R	44	5.84	44	8	30
	R	47	4.92	38	30	0
medial prefrontal gyrus	L	8	6.23	−2	38	54
	R	9	5.08	6	62	32
anterior cingulate/medial prefrontal	L	9/32	5.26	−12	46	16
cingulate gyrus		24	4.83	0	6	34
amygdala	L		6.15	−22	−2	−24
	R		6.73	18	0	−20
hippocampus	L		5.42	−28	−14	−16
	R		5.09	30	−12	−16
temporal pole	L		6.08	−32	12	−38
precentral gyrus	L	6	4.94	−60	−26	36
	R	6	5.51	66	−22	38
inferior parietal cortex	R	40	5.06	32	−46	52
superior parietal cortex	L	7	5.14	−24	−54	54
fusiform gyrus/inferior occipital gyrus	L	37/20	Inf	−46	−80	−6
	R	37/19	Inf	52	−62	−10
lingual gyrus	R	19	4.92	26	−66	−2
cuneus/occipital gyrus	L	18/19	6.06	−26	−76	26
superior occipital gyrus	R	19	5.12	30	−74	30
brainstem	L		7.14	−6	−26	−8
	R		7.26	8	−26	−8
cerebellum	R		6.09	2	−58	−44
*Group activation for regulation>no regulation*						
dorsolateral prefrontal cortex	R	9	5.88	40	26	44
superior frontal gyrus	R	6	5.21	22	14	62
inferior parietal cortex	L	40	5.43	−62	−46	40
	R	40	7.13	58	−54	40
*Group activation for no regulation>regulation*						
amygdala	L		4.09*	−20	−2	−26
	R		4.80	22	2	−26
hippocampus	L		6.04	−22	−28	−4
	R		5.16	26	−26	−6
parahippocampal gyrus	L	28/35	5.48	−26	−20	−18
	R	28/35	5.18	22	−22	−14
cuneus	L	31	4.92	−10	−60	8
	R	31	4.87	14	−68	12
fusiform/inferior occipital gyrus	L	18	7.84	−12	−100	−2
	R	18	7.84	18	−96	−6
lingual gyrus	R	19	5.16	10	−52	2
*Group activation for negative regulation>negative no regulation*						
dorsolateral prefrontal cortex	R	9	6.11	40	26	44
superior frontal gyrus	R	6	4.89	22	14	64
inferior parietal cortex	L	40	4.98	−62	−46	40
	R	40	6.83	58	−54	40
*Group activation for neutral regulation>neutral no regulation*						
inferior parietal cortex	R	40	6.16	50	−48	40
*Group activation for negative no regulation>negative regulation*						
amygdala	L		3.60*	−20	−10	−10
	R		3.85*	18	−6	−20
hippocampus	L		5.82	−22	−28	−4
parahippocampal gyrus	L	28/35	5.19	−26	−20	−18
occipital/fusiform gyrus	L	17/18	7.80	−26	−86	−14
	R	17/18	7.23	18	−96	−6
brainstem			4.79	−6	−30	−8
			5.34	10	−28	−8
cerebellum			4.74	−20	−58	−18
			5.10	28	−46	−24
*Group activation for neutral no regulation>regulation*						
amygdala	L		3.53*	−20	−2	−26
	R		3.94*	24	−8	−20
hippocampus	L		5.02	−22	−30	−4
parahippocampal gyrus	R	28/35	4.92	22	−22	−14
occipital/fusiform gyrus	L	18	7.29	−26	−98	−2
lingual gyrus	R		4.69	12	−52	2
cerebellum	R		4.73	46	−66	−24

All results: p<0.05 FWE corrected for multiple comparisons; ^*^p<0.05 FWE corrected for region of interest; BA Brodmann area; x,y,z, respective coordinates of MNI template.

To identify whether the level of regulation related increase of dorsolateral prefrontal (DLPFC) activation was correlated with the regulation related decrease in amygdala activation we performed a regression analysis on the extracted individual data for the contrast regulation negative>no-regulation negative (DLPFC) with the contrast no-regulation negative>regulation negative (amygdala left and right). We found a significant positive correlation between activation increase in right DLPFC and activation decrease in the left (r = 0.66, p = 0.0014) (Fig. 1) as well as the right amygdala (r = 0.51, p = 0.015).

In order to test for an immediate aftereffect in both amygdala we extracted the averaged time series in each subject (see Methods section). While amygdala activation was effectively reduced *during* intentional regulation it subsequently increased in the postregulational relax period (Fig. 1). This effect was significant for the regulated negative but not for the regulated neutral condition (interaction of negative regulation×period (scan 5 resp. 9, see methods): left amygdala F(1,17) = 17.34; p = 0.00065; right amygdala F(1,17) = 5.43; p = 0.04; interaction of neutral regulation×period: left amygdala F(1,17) = 0.9; p = 0.4; right amygdala F(1,17) = 0.36; p = 0.6).

Regarding correlations of habitual emotion regulation (ERQ) and WBSI with the immediate aftereffect, we found a significant positive correlation between the individual WBSI score and the peak magnitude (difference score between scan 9 regulation and scan 9 no regulation) of the immediate aftereffect in the left amygdala (r = 0.48; p = 0.02 (Fig. 1).

**Figure 2:**
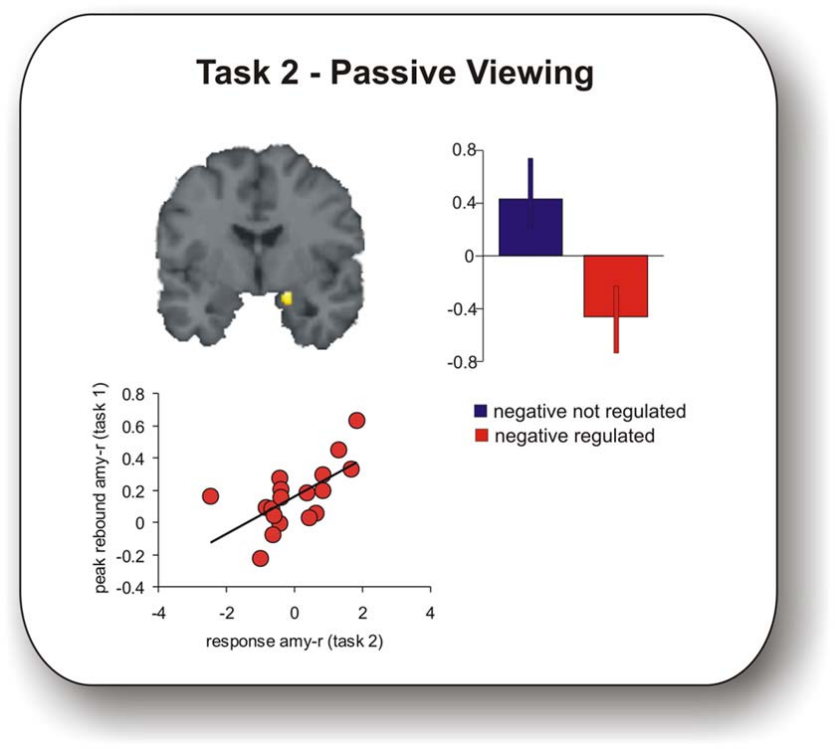
Task 2 (passive viewing). Upper row: Sustained downregulation of amygdala activation for formerly regulated negative pictures (p<0.05 FWE corrected for ROI; this effect was also significant for the left amygdala (not shown here)). Bottom row: Amygdala activation during presentation of formerly regulated negative pictures in task 2 correlated positively with individual differences in peak rebound activation in task 1 (p<0.05, FWE corrected for ROI; this effect was also significant at a lower statistical level (p = 0.008 uncorrected) for the left amygdala (not shown here)).

*Task 2 (passive viewing)*: For detailed results please see Table 2. Presentation of negative pictures elicited a significant activation in the amygdala bilaterally compared to neutral pictures (main effect of emotion). However, activation in response to formerly regulated negative pictures was significantly reduced (Fig. 2). This effect was significant also in an interaction of valence and former regulation (Tab. 2). We observed no main effect of former regulation on the prefronto-parietal network as in task 1, i.e. increased activation during presentation of pictures formerly regulated, even when lowering the threshold to an uncorrected level of p<0.05.

**Table 2:** Results of Task 2 (Passive Viewing).

Region		BA	Z	x	y	z
*Group activation for negative>neutral*						
inferior frontal gyrus	L	47	4.78	−46	24	−2
amygdala	L		3.53*	−18	−6	−18
fusiform/occipital gyrus	L	19/37	5.23	−46	−76	−12
	R	19/37	4.88	54	−64	−14
*Group activation for no regulation>regulation*						
lingual gyrus	L	18	5.34	−12	−94	−4
*Group activation for negative no regulation>negative regulation*						
lingual gyrus	L	18	5.23	−16	−86	−18
amygdala	L		4.06*	−20	−8	−12
	R		3.00*	20	0	−18
*Group activation for negative (no regulation>regulation)>neutral (no regulation>regulation)*						
amygdala	L		3.35*	−20	−10	−12
	R		3.17*	20	−2	−14

All results: p<0.05 FWE corrected for multiple comparisons; ^*^p<0.05 FWE corrected for region of interest; BA Brodmann area; x,y,z, respective coordinates of MNI template.

To explore the relation of both aftereffects, a simple regression analysis was performed, testing whether amygdala activation during presentation of formerly regulated negative pictures in task 2 (passive viewing) covaried with individual differences in peak immediate aftereffect magnitude (taken from scan 9 similar to the procedure in the interaction analysis of time courses) in task 1. We observed a positive correlation between right amygdala activation to formerly regulated negative pictures in task 2 and the peak magnitude of the ipsilateral immediate aftereffect following offset of regulated negative pictures in task 1 (p<0.05, FWE corrected for ROI, x = 20, y = −2 z = −14) (Fig. 2). A similar correlation was found for the left amygdala between task 1 and 2 at p = 0.008 uncorrected for multiple comparisons (x = −18, y = −4, z = −18). There were no correlations between the second aftereffect and habitual emotion regulation or WBSI scores.

## Discussion

In the present study, we found, as expected, that voluntary emotion regulation by detachment was highly effective in reducing amygdala activation during active regulation. Moreover, we found two aftereffects, which were related to each other: We observed a delayed aftereffect with a sustained decrease of amygdala activation after 10 minutes that was related to an immediate aftereffect i.e. an increase of amygdala activation directly following stimulus offset.

### Effects during intentional emotion regulation

All subjects were able to successfully regulate their feelings upon presentation of negative pictures as evidenced by debriefing of subjects and our control experiment. Consistent with other studies [10], [14], [16]–[18] regulation was accompanied by activation of a right hemispheric regulation network as well as by a reduction of amygdala activation *during* regulation. Moreover, the amount of decrease in amygdala activation was positively correlated with the regulation related increase in right DLPFC activation, suggesting a top-down inhibitory effect of DLPFC on amygdala function, which is consistent with the literature on voluntary emotion regulation [3], [20]. As the DLPFC has only sparse anatomical connections to the amygdala [37], this effect is most likely mediated by a circuit involving medial parts of the prefrontal cortex [23]. However, this observed correlation observed certainly does not prove causation.

### Sustained regulation effect

Ten minutes after termination of the active regulation task (task 1), we still observed a sustained downregulation of amygdala in task 2 (passive viewing) for those negative items that were formerly regulated. In other words, emotion regulation is not only effective online, but extends at least ten minutes after termination of active regulation. Is this a mere repetition effect? It is well known that a second view of the same emotional stimuli might lead to less activation in the amygdala (e.g. [38]–[40]). However, we can exclude this possibility, as a mere repetition effect would be apparent in regulated as well as non-regulated pictures. Alternatively, one might argue that subjects again voluntarily used a detachment procedure while viewing the respective pictures in the second task. However, we did not give any instructions to regulate, subjects did not report that they did, and randomized order and short presentation time of the pictures make it unlikely that they were able to. Moreover, we did not find activation of the fronto-parietal network in the respective condition, not even at an uncorrected level of p<0.05. Therefore, we assume that the sustained regulation effect is a real effect of former regulation, i.e. that active regulation through the DLPFC in task 1 has prepared the brain to react less intense to negative stimuli when they appear later in random order for a very short time. The DLPFC is crucially involved in the implementation of associations between context and adaptive behaviour [41], [42]. Therefore, one might speculate that a potential mechanism for the observed sustained regulation effect in the amygdala is caused by a DLPFC-initiated remodelling of stimulus-response-associations so that it is no longer necessary to mobilize resources of effortful control. Another possible explanation for this effect may be that the regulation process changes the meaning of each specific regulated stimulus for the individual, although it is an open question where or how meaning is changed and subsequently stored – here the DLPFC might be involved in changing stimulus meaning, while other neural circuits (possibly more posterior regions) might be responsible for storing specific stimulus meanings. A further possible explanation for the sustained effect of regulation is that the picture serves as a cue or prime for the specific meaning or story the participant attached to the respective picture during the regulation task 1. In this instance, it is the generated story or meaning, rather than the picture itself which determines the amygdala response – an explanation that fits well with the ideas of appraisal theories of emotion. However, these assumptions are only speculative and should be further investigated in future studies.

### Immediate paradoxical aftereffect

We tested for immediate aftereffects during the relaxation period in task 1 by analysing the signal time course in the amygdala. For non-regulated items, the amygdala signal reached a peak during picture presentation and declined in the relaxation period (Fig. 1, blue line). During active regulation, the amygdala signal was significantly reduced, however, paradoxically increased during the respective relaxation period (Fig. 1, red line).

Does this effect simply reflect picture offset? No, because then the signal should likewise increase following non-regulated items which was not the case. Another explanation might be that subjects consciously ruminate about the previously downregulated content of the picture. Although we cannot definitely exclude this possibility it is rather unlikely, because 14 out of 18 subjects reported in the debriefing that they did not think about the pictures in the relaxation period. A third explanation, which we would like to suggest, is that the increase in amygdala activation signifies a paradoxical rebound effect. Rebound effects have been described in other domains and at different time scales before – for example in the field of thought suppression [28], [31]. Clearly, voluntary emotion regulation taps other processes than thought suppression and happens on different time scales. However, a common aspect here is that there is a paradoxical effect delayed in time. A further alternative explanation for this delay is that what we call rebound here is a shift in peak amygdala activation induced by detachment. As we did not vary the duration of picture presentation and thus regulation period, we cannot distinguish between both interpretations. This should be investigated in further studies.

Our data further point to the possibility that emotion regulation by detachment and thought suppression might not be totally independent, as we found that the peak amygdala activation during regulation correlated positively with individual scores in the WBSI, suggesting that subjects with greater habitual thought control have a higher postregulational response. On a psychophysiological level similar findings have been reported in thought suppression experiments showing elevated electrodermal responses after suppression of arousing thoughts in subjects scoring high in the WBSI [33]. However, in order to disentangle the relations between emotion regulation by detachment and thought suppression on the neural level [43] it is necessary to investigate and directly compare both strategies in a follow up-experiment.

### Modulation of the sustained by the immediate aftereffect

Interestingly, the immediate paradoxical aftereffect modulated the sustained regulation effect: The higher the postregulational increase in amygdala signal the smaller was the sustained regulation effect (Fig. 1). Although we cannot draw any firm conclusion from this correlational pattern, this relation suggests that there is a physiological surrogate marker for the efficiency of sustained emotion regulation. It will be of great interest to further explore the mechanisms underlying these observations.

### Limitations

One limitation of our study is that we did not use online ratings in the fMRI study. However, as explained, this was essential to our design as we intended to avoid self-referential cognitive evaluation during the relexation period. Our control experiment shows that our design works well. Furthermore, we did not use psychophysiological indicators of emotional involvement, like skin conductance response, which would allow us to judge the success of emotion regulation as an additional dependent variable. Also, we did not register eye movements, in order to control for fixation. Finally, in our study we investigated only women, in order to rule out effects of gender, therefore our inferences and conclusions cannot be generalized to male subjects.

### Conclusion

In summary, we have shown that voluntary emotion regulation extends beyond the period of emotion regulation itself. The most important finding from a clinical perspective is that there is sustained downregulation of amygdala activation even if no active regulation is employed and no neural indication of active regulation is apparent. As emotion regulation normally is intended to be effective for longer time periods, the investigation of aftereffects can be used for evaluation of the effectiveness of different emotion regulation strategies from a neurobiological perspective. Also, it is possible and would be interesting to explore aftereffects on larger time scales, i.e. from days to months.

Moreover, we could demonstrate that sustained regulation is modulated by several factors. In particular, the immediate paradoxical aftereffect, interpreted by us as a rebound effect, diminishes the effectiveness of sustained regulation. If it will turn out that the immediate aftereffect is indicative for sustained regulation, it could be used for prediction of longer term regulation effects on the brain. Additionally, we found that habitual thought suppressors show a larger immediate aftereffect. At this point, the relationship between emotion regulation and thought suppression is only descriptive and is still opaque. Further studies are needed to better elucidate how both phenomena are related to one another.

As the next step, we suggest to directly compare different emotion regulation strategies, like cognitive reinterpretation, suppression of emotion expression, and thought suppression with detachment. Moreover, it will be of interest to investigate whether and to what extent aftereffects differ for subjects suffering from affective disorders or posttraumatic stress disorders. Finally, since genetic variation has an impact on the reactivity of the amygdala [44], [45] it will be of interest to study influences of common polymorphisms on aftereffects.

## References

[1] Frijda N (1986). The Emotions..

[2] Davidson RJ, Pizzagalli D, Nitschke JB, Putnam K (2002). Depression: perspectives from affective neuroscience.. Annu Rev Psychol.

[3] Phillips ML, Ladouceur CD, Drevets WC (2008). A neural model of voluntary and automatic emotion regulation: implications for understanding the pathophysiology and neurodevelopment of bipolar disorder.. Mol Psychiatry.

[4] Freud S (1926). Hemmung, Symptom und Angst..

[5] Lazarus RS (1966). Psychological stress and the coping process..

[6] Gross JJ (2002). Emotion regulation: affective, cognitive, and social consequences.. Psychophysiology.

[7] Jackson DC, Malmstadt JR, Larson CL, Davidson RJ (2000). Suppression and enhancement of emotional responses to unpleasant pictures.. Psychophysiology.

[8] Ochsner KN, Gross JJ (2005). The cognitive control of emotion.. Trends Cogn Sci.

[9] Ochsner KN, Gross JJ (2008). Cognitive Emotion Regulation: Insights from Social Cognitive and Affective Neuroscience.. Current Directions in Psychological Science.

[10] Hariri AR, Mattay VS, Tessitore A, Fera F, Weinberger DR (2003). Neocortical modulation of the amygdala response to fearful stimuli.. Biol Psychiatry.

[11] Erk S, Abler B, Walter H (2006). Cognitive modulation of emotion anticipation.. Eur J Neurosci.

[12] Erk S, Kleczar A, Walter H (2007). Valence-specific regulation effects in a working memory task with emotional context.. Neuroimage.

[13] Beauregard M, Levesque J, Bourgouin P (2001). Neural correlates of conscious self-regulation of emotion.. J Neurosci.

[14] Eippert F, Veit R, Weiskopf N, Erb M, Birbaumer N (2007). Regulation of emotional responses elicited by threat-related stimuli.. Hum Brain Mapp.

[15] Levesque J, Eugene F, Joanette Y, Paquette V, Mensour B (2003). Neural circuitry underlying voluntary suppression of sadness.. Biol Psychiatry.

[16] Goldin PR, McRae K, Ramel W, Gross JJ (2008). The neural bases of emotion regulation: reappraisal and suppression of negative emotion.. Biol Psychiatry.

[17] Ochsner KN, Bunge SA, Gross JJ, Gabrieli JD (2002). Rethinking feelings: an FMRI study of the cognitive regulation of emotion.. J Cogn Neurosci.

[18] Phan KL, Fitzgerald DA, Nathan PJ, Moore GJ, Uhde TW (2005). Neural substrates for voluntary suppression of negative affect: a functional magnetic resonance imaging study.. Biol Psychiatry.

[19] Kalisch R, Wiech K, Critchley HD, Seymour B, O'Doherty JP (2005). Anxiety reduction through detachment: subjective, physiological, and neural effects.. J Cogn Neurosci.

[20] Kalisch R, Wiech K, Herrmann K, Dolan RJ (2006). Neural correlates of self-distraction from anxiety and a process model of cognitive emotion regulation.. J Cogn Neurosci.

[21] Ochsner KN, Knierim K, Ludlow DH, Hanelin J, Ramachandran T (2004). Reflecting upon feelings: an fMRI study of neural systems supporting the attribution of emotion to self and other.. J Cogn Neurosci.

[22] Urry HL, van Reekum CM, Johnstone T, Kalin NH, Thurow ME (2006). Amygdala and ventromedial prefrontal cortex are inversely coupled during regulation of negative affect and predict the diurnal pattern of cortisol secretion among older adults.. J Neurosci.

[23] Banks SJ, Eddy KT, Angstadt M, Nathan PJ, Phan KL (2007). Amygdala-frontal connectivity during emotion regulation.. Soc Cogn Affect Neurosci.

[24] Beauregard M, Paquette V, Levesque J (2006). Dysfunction in the neural circuitry of emotional self-regulation in major depressive disorder.. Neuroreport.

[25] Johnstone T, van Reekum CM, Urry HL, Kalin NH, Davidson RJ (2007). Failure to regulate: counterproductive recruitment of top-down prefrontal-subcortical circuitry in major depression.. J Neurosci.

[26] Goldin PR, Manber T, Hakimi S, Canli T, Gross JJ (2009). Neural bases of social anxiety disorder: emotional reactivity and cognitive regulation during social and physical threat.. Arch Gen Psychiatry.

[27] Freud S (1915). Die Verdrängung. Psychologie des Unbewussten..

[28] Abramowitz JS, Tolin DF, Street GP (2001). Paradoxical effects of thought suppression: a meta-analysis of controlled studies.. Clin Psychol Rev.

[29] Koster EH, Rassin E, Crombez G, Naring GW (2003). The paradoxical effects of suppressing anxious thoughts during imminent threat.. Behav Res Ther.

[30] Roemer L, Borkovec TD (1994). Effects of Suppressing Thoughts About Emotional Material.. Journal of Abnormal Psychology.

[31] Wenzlaff RM, Wegner DM (2000). Thought suppression.. Annu Rev Psychol.

[32] Gross JJ, John OP (2003). Individual differences in two emotion regulation processes: implications for affect, relationships, and well-being.. J Pers Soc Psychol.

[33] Wegner DM, Zanakos S (1994). Chronic thought suppression.. J Pers.

[34] Hariri AR, Bookheimer SY, Mazziotta JC (2000). Modulating emotional responses: effects of a neocortical network on the limbic system.. Neuroreport.

[35] Eickhoff SB, Stephan KE, Mohlberg H, Grefkes C, Fink GR (2005). A new SPM toolbox for combining probabilistic cytoarchitectonic maps and functional imaging data.. Neuroimage.

[36] Ochsner KN, Ray RD, Cooper JC, Robertson ER, Chopra S (2004). For better or for worse: neural systems supporting the cognitive down- and up-regulation of negative emotion.. Neuroimage.

[37] Ghashghaei HT, Barbas H (2002). Pathways for emotion: interactions of prefrontal and anterior temporal pathways in the amygdala of the rhesus monkey.. Neuroscience.

[38] Ishai A, Pessoa L, Bikle PC, Ungerleider LG (2004). Repetition suppression of faces is modulated by emotion.. Proc Natl Acad Sci U S A.

[39] Fischer H, Wright CI, Whalen PJ, McInerney SC, Shin LM (2003). Brain habituation during repeated exposure to fearful and neutral faces: a functional MRI study.. Brain Res Bull.

[40] Wright CI, Fischer H, Whalen PJ, McInerney SC, Shin LM (2001). Differential prefrontal cortex and amygdala habituation to repeatedly presented emotional stimuli.. Neuroreport.

[41] Bunge SA (2004). How we use rules to select actions: a review of evidence from cognitive neuroscience.. Cogn Affect Behav Neurosci.

[42] Miller EK, Cohen JD (2001). An integrative theory of prefrontal cortex function.. Annu Rev Neurosci.

[43] Mitchell JP, Heatherton TF, Kelley WM, Wyland CL, Wegner DM (2007). Separating sustained from transient aspects of cognitive control during thought suppression.. Psychol Sci.

[44] Hariri AR, Drabant EM, Weinberger DR (2006). Imaging genetics: perspectives from studies of genetically driven variation in serotonin function and corticolimbic affective processing.. Biol Psychiatry.

[45] Hariri AR, Holmes A (2006). Genetics of emotional regulation: the role of the serotonin transporter in neural function.. Trends Cogn Sci.

